# Prognostic Significance of Platelet-to-Lymphocyte Ratio in Cholangiocarcinoma: A Meta-Analysis

**DOI:** 10.1155/2018/7375169

**Published:** 2018-11-14

**Authors:** Gang Hu, Qin Liu, Jian-ying Ma, Cheng-yuan Liu

**Affiliations:** ^1^Department of Breast Surgery, Thyroid Surgery, Pancreatic Surgery, Huangshi Central Hospital of Edong Healthcare Group, Hubei Polytechnic University, No. 141, Tianjin Road, Huangshi, Hubei, China; ^2^Department of Emergency Surgery, The First Affiliated Hospital of Nanchang University, No. 17 Yongwai Street, Nanchang, Jiangxi Province 330006, China

## Abstract

**Introduction:**

Pretreatment platelet-to-lymphocyte ratio (PLR) has been considered a prognostic factor in various cancers. However, the application of PLR in the assessment of patients with cholangiocarcinoma remains controversial. This study aimed to evaluate the prognostic value of pretreatment PLR in cholangiocarcinoma.

**Methods:**

A systematic search was performed in MEDLINE, EMBASE, and Cochrane Library to identify studies assessing the prognostic significance of the pretreatment PLR in cholangiocarcinoma. Three databases were searched from inception to August 5, 2018. The primary outcome was overall survival (OS), and the secondary outcomes were recurrence-free survival (RFS) and progression-free survival (PFS). Pooled hazard ratios (HRs) or odds ratios (ORs) with 95% confidence intervals (CIs) were calculated using random-effects models.

**Results:**

A total of 9 studies including 2395 patients were finally enrolled in the meta-analysis based on the inclusion and exclusion criteria. All of the included studies were retrospective observational cohorts. Elevated PLR predicted poor OS (HR: 1.38, 95% CI: 1.19-1.62, P < 0.001) and RFS or PFS (HR = 1.55; 95% CI = 1.27-1.88; P < 0.001). Moreover, elevated PLR was highly associated with male sex (male versus female OR = 0.59, 95% CI: 0.44-0.80, P < 0.001) and R1 resection margin (OR = 2.09, 95% CI: 1.24-3.54, P = 0.006).

**Conclusion:**

The present meta-analysis demonstrated that pretreatment PLR might serve as a useful prognostic biomarker in cholangiocarcinoma.

## 1. Introduction

Cholangiocarcinoma (CCA) is a primary liver cancer with features of differentiation of cholangiocytes, the epithelial cells lining the intra- and extrahepatic portions of the biliary tree [1]. An increasing incidence of CCA has been reported over the last few decades [2]. It is the second most frequent type of primary liver cancer and comprises malignancies with high inter- and intratumor heterogeneities. It is currently classified into intrahepatic, perihilar, and distal extrahepatic cholangiocarcinoma [3]. Surgical resection remains the best therapeutic approach for CCA, but unfortunately most patients are diagnosed at an unresectable stage of the disease. Although the accuracy of current diagnostic methods has greatly improved, the 5-year overall survival (OS) remains poor [4, 5]. Therefore, a reliable and readily accessible preoperative prognostic biomarker is required to determine the optimal therapeutic strategies.

A growing number of studies have shown that cancer-related inflammation results in poor prognosis. Moreover, inflammation plays a strong role in tumor development, progression, and metastasis [6]. Accordingly, inflammation-based prognostic indicators, such as the Glasgow prognostic score (GPS), C-reactive protein (CRP), and neutrophil-to-lymphocyte ratio (NLR), have been investigated in various cancers [7–9]. The NLR has been associated with worse prognosis in various cancers [10–12]. However, because of the inconsistent results, whether PLR is associated with the prognosis in CCA remains controversial [13–15]. We therefore conducted a meta-analysis to assess the prognostic role of PLR and analyze the relationships between PLR and clinicopathological parameters in patients with CCA.

## 2. Materials and Methods

### 2.1. Search Strategies

A systematic search of electronic databases, including MEDLINE, EMBASE, and Cochrane Library, was performed up to August 5, 2018, to obtain relevant articles for the meta-analysis. Studies were selected using the following key words: “cholangiocarcinoma” or “bile duct cancer” and “tumor” or “cancer” or “neoplasm” or “carcinoma” or “malignancy” and “platelet lymphocyte ratio” or “PLR”. Other relevant studies were also obtained by manually screening the references list.

### 2.2. Selection Criteria

The inclusion criteria were as follows: (1) studies investigate the PLR and survival in CCA; (2) CCA was confirmed by pathological examination; (3) the HR and 95% CI, or Kaplan–Meier survival curves from which an HR could be calculated, were reported; and (4) a cut-off value for PLR was reported. The exclusion criteria were as follows: (1) reviews, letters, or conference abstracts; (2) insufficient data or unavailable data; and (3) studies with duplicate data.

### 2.3. Data Extraction and Quality Assessment

Two investigators (G.H. and Q.L.) performed the data extraction independently. Data were extracted as follows: first author's name, publication year, country, number of patients, follow-up period, treatment, gender, age, CA199, differentiation, lymph node metastasis, vascular invasion, postoperative complication, postoperative mortality, margin status, survival analysis methods, HR estimate, and cut-off values. Margin status included R0 (microscopically negative resection margins) and R1 (microscopically positive resection margins).

The methodological quality of included studies was independently assessed by two independent reviewers (G.H. and Q.L.) according to the Newcastle-Ottawa Scale (NOS) [16], which included three primary domains: selection, comparability, and outcome. Studies with an NOS score of *⩾*6 were deemed high-quality studies. Any discrepancy was resolved by joint discussion.

### 2.4. Statistical Analysis

We used Stata 13.0 statistical software (Stata, College Station) to estimate HRs for OS, PFS, and RFS and odd ratios (ORs) for clinicopathological parameters. If the statistical variables were described in the study, we extracted them directly. Otherwise, they were calculated with Kaplan-Meier survival curves, which were read according to the methods described by Tierney et al. and Parmar [17, 18]. The heterogeneity among the studies was evaluated by the chi-square value and the I^2^ value. If I^2^ ⩽ 50% or P > 0.05, a fixed-effects model was used for analysis. If not (I^2^ > 50% or P ⩽ 0.05), a random-effects model was used. We then performed subgroup analyses to examine the potential source of heterogeneity. To validate the credibility of the result, sensitivity analyses were performed by removing each study. A P value less than 0.05 was considered statistically significant.

## 3. Results

### 3.1. Study Characteristics

**Figure 1:**
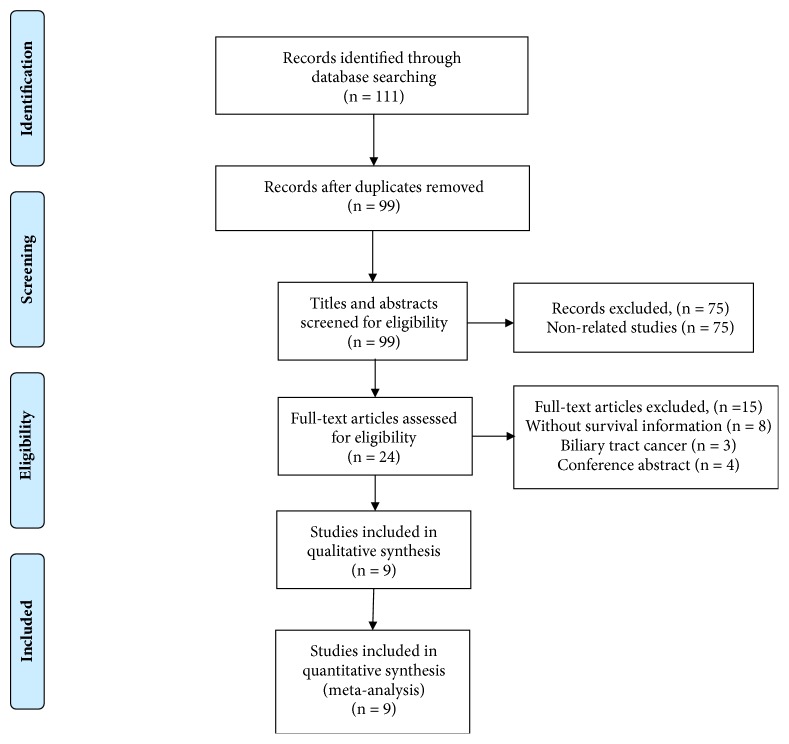
The flow chart of study selection procedure in the meta-analysis.

As shown in the flow diagram (Figure 1), 111 potentially relevant articles were obtained through electronic searches. 99 articles remained after exclusion of duplicated data. After screening the titles and abstracts carefully, 75 articles were excluded. Finally, a total of 9 studies were included in the meta-analysis [13–15, 19–24]. All of the included studies were retrospective observational cohorts. Most of these studies have been published since 2017. Of the 9 studies, four studies were from China, three were from Japan, one was from Korea, and one was from multiple centers. The treatments were surgery and mixed methods. All studies assessed the association between pretreatment PLR and OS, whereas 4 studies reported RFS or PFS. Cut-off values of PLR ranged from 123 to 190. The main characteristics of the 9 enrolled studies are shown in Table 1. NOS scores of all the studies were at least 6 or more (Table 2).

### 3.2. Meta-Analysis

#### 3.2.1. Impact of PLR on OS

**Figure 2:**
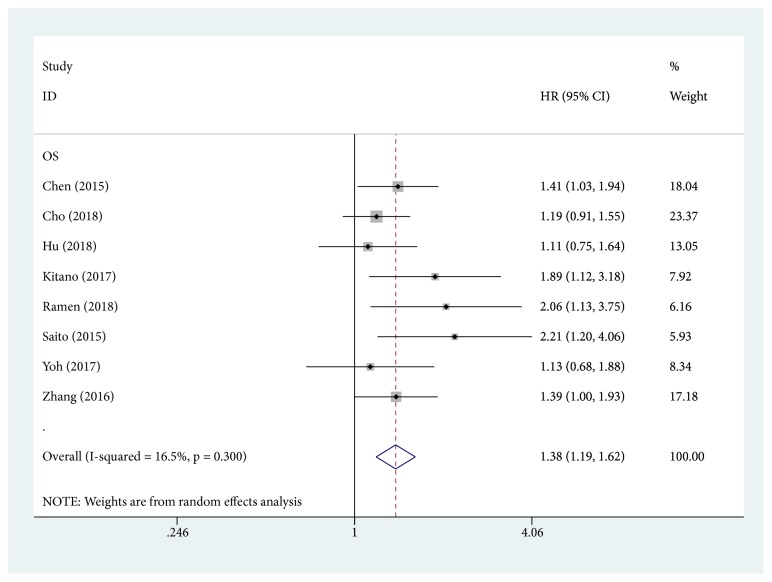
Forest plots for the association between PLR and OS.

Nine studies, comprising 2395 patients, reported the relationship between PLR and OS. The HR, expressed as the high-PLR group versus the low-PLR group, was 1.00 (95% CI = 1.00-1.00, P = 0.085). Buettner et al.'s study was not included in this meta-analysis of OS. The pooled result showed that patients with high PLR had a worse OS (HR: 1.38, 95% CI: 1.19-1.62, P < 0.001), with no heterogeneity (I^2^= 16.5%, P = 0.30; Figure 2). The association between PLR and OS was further evaluated by subgroup analysis based on the main features, including tumor stage, cut-off for PLR, treatment, and analysis method (Table 3). The results indicated that elevated PLR significantly predicted shorter OS in patients who received surgery (HR = 1.43; 95% CI = 1.12-1.83; P = 0.005) or mixed treatments (HR = 1.89; 95% CI = 1.11-3.14; P = 0.020). When stratified by disease stage, PLR was a prognostic factor in patients with mixed stages (HR = 1.40; 95% CI = 1.18-1.67; P < 0.001). Pooled HRs for OS were stratified by HR analysis methods. The negative effect of elevated PLR on OS was observed by multivariate analysis (HR = 1.52; 95% CI = 1.27-1.81; P < 0.001). Moreover, PLR showed prognostic value regardless of the cut-off value for NLR (*⩾* 150 and < 150).

#### 3.2.2. Impact of PLR on PFS/RFS

**Figure 3:**
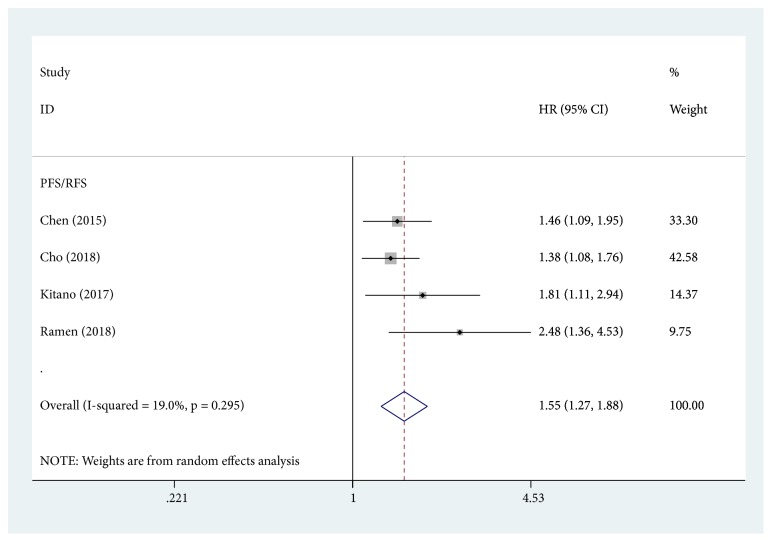
Forest plots for the association between PLR and PFS/RFS.

Four studies were included in the analysis of PLR and PFS/RFS. The pooled HR was 1.55, which indicated that elevated PLR was significantly associated with poor PFS/RFS (Figure 3). There was no significant heterogeneity between the included studies (I^2^ = 19.0%; P = 0.295).

#### 3.2.3. Associations between PLR and Clinicopathological Parameters

To further exploit the impact of PLR on clinicopathological features, we identified 9 clinicopathological parameters (Table 4). As shown in Table 3, the results demonstrated that elevated PLR was highly correlated with gender (male versus female; OR = 0.59, 95% CI: 0.44-0.80, P < 0.001) and margin status (R1 versus R0; OR = 2.09, 95% CI: 1.24-3.54, P = 0.006). However, elevated PLR was not related to age (*⩾* 45 versus < 45; OR = 0.82, 95% CI: 0.38-1.77, P = 0.61), CA199 (>37 ng/mL versus <37 ng/mL; OR = 1.25, 95% CI: 0.92-1.70, P = 0.16), differentiation (low versus moderate/high; OR = 1.05, 95% CI: 0.64-1.73, P = 0.85), lymph node metastasis (pos versus neg; OR = 1.16, 95% CI: 0.82-1.65, P = 0.39), vascular invasion (pos versus neg; OR = 1.27, 95% CI: 0.86-1.89, P = 0.23), postoperative complications (present versus absent; OR = 1.44, 95% CI: 0.97-2.14, P = 0.07), and postoperative mortality (present versus absent; OR = 1.54, 95% CI: 0.56-4.26, P = 0.41).

### 3.3. Sensitivity Analysis

**Figure 4:**
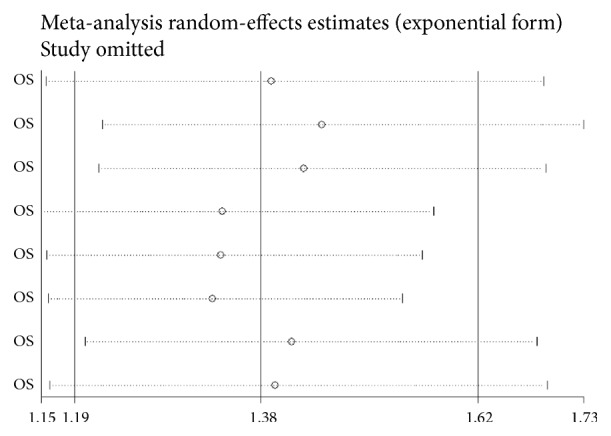
Sensitivity analysis of PLR on OS in CCA patients.

Sensitivity analysis was performed to assess the stability of the results. The result was not significantly impacted by removing any eligible study (Figure 4).

## 4. Discussion

In this study, a meta-analysis was conducted to investigate the correlations between pretreatment PLR and clinicopathological characteristics and to evaluate the prognostic value of PLR in patients with CCA. The combined results demonstrated that elevated PLR is significantly associated with worse OS and RFS/PFS. Therefore, PLR could serve as biomarker for the prognosis of CCA patients. Additionally, the correlations between PLR and clinicopathological parameters were evaluated. Elevated PLR was correlated with female sex and margin status (R1).

The exact mechanisms by which PLR predicts poor outcome of CCA patients are still undefined. Emerging evidence has indicated strong linkage between systemic inflammatory response and tumor development [6, 25, 26]. Platelets, as a participant in the inflammatory response, protect tumor cells from natural killer-mediated lysis, thus supporting the tumor metastasis [27]. A variety of platelet-associated chemokines can modulate inflammation within the tumor environment and tumor angiogenesis, such as platelet factor 4 (PF-4/CXCL4) and connective tissue-activating peptide III (CTAP-III) [28]. Lymphocytes play a major role in suppressing cancer cell proliferation and migration [29]. Tumor-infiltrating lymphocytes (TILs) are vital components of the antitumor immune microenvironment and are involved in several stages of tumor progression [30, 31]. Tumor-infiltrating CD8+ and CD4+ T lymphocytes induce cytotoxic cell death and inhibit tumor cell proliferation and migration in antitumor immune reactions [32, 33]. Conversely, low lymphocyte counts may lead to inadequate immune responses, resulting in poor survival of many cancers [34, 35]. Thus, PLR may represent a balance between the tumor promotion reaction and antitumor immune function.

Several limitations should be taken into consideration when interpreting our findings. First, the cut-off value of PLR applied in the enrolled studies was not uniform. Second, all of the included studies were retrospective and published in English. Third, this meta-analysis is not registered online. Fourth, all included studies were from Asia, which means that our data do not represent the CCA picture globally. It remains unclear whether these findings might be applied to other populations. Therefore, more large-scale studies are warranted to assess the prognostic value of pretreatment PLR for cervical cancer patients.

## 5. Conclusions

Our meta-analysis confirmed that elevated pretreatment PLR is associated with poor prognosis in patients with CCA. Therefore, PLR may serve as a promising biomarker for predicting prognosis in patients with CCA.

## Figures and Tables

**Table 1:** Characteristics of the studies included in the meta-analysis.

Author	Year	Area	Follow-up (months)	Treatment	No. of patients	Stage	Cut-off value	Survival analysis	Analysis
Buettner	2018	Multicenter	29 (4.8-53.3)	Surgery	991	NA	190	OS	UV
Chen	2015	China	57.8±11.2	Surgery	322	Mixed	123	OS/RFS	MV
Cho	2018	Korea	25 (19.6-30.4))	Chemotherapy	257	Metastatic	123.8	OS/PFS	UV
Hu	2018	China	NA	Surgery	173	Mixed	150	OS	UV
Kitano	2017	Japan	NA	Mixed	120	Mixed	185	OS/RFS	MV
Ramen	2018	China	NA	Surgery	90	NA	148	OS/RFS	MV
Saito	2015	Japan	70 (42-82)	Surgery	121	Mixed	150	OS	MV
Yoh	2017	Japan	65 (26-84)	Surgery	134	Mixed	120	OS	MV
Zhang	2016	China	NA	NA	187	Mixed	138	OS	MV

OS: overall survival; PFS: progress-free survival; RFS: recurrence-free survival; UV: univariate; MV: multivariate; NA: not available.

**Table 2:** Assessment of study quality.

Author	Selection	Comparability	Outcome	Total score
Buettner	★★★★	★★	★★★	9
Chen	★★★★	★★	★★	8
Cho	★★★★	★★	★★	8
Hu	★★★	★	★★	6
Kitano	★★★★	★	★	6
Ramen	★★★	★★	★★★	8
Saito	★★★★	★	★	6
Yoh	★★★★	★	★★	7
Zhang	★★★★	★	★	6

**Table 3:** Pooled hazard ratios (HRs) for OS according to subgroup analyses.

Subgroup	No. of studies	No. of patients	HR (95% CI)	P value	Heterogeneity	
					I^2^(%)	P_h_
Overall	8	1404	1.38 (1.19-1.62)	<0.001	16.5	0.30
Treatment						
Surgery	5	840	1.43 (1.12-1.83)	0.005	30.8	0.22
Chemoradiotherapy	1	257	1.19 (0.91-1.55)	0.200	—	—
Mixed	1	120	1.89 (1.11-3.14)	0.020	—	—
Stage						
Mixed	6	866	1.40 (1.18-1.67)	<0.001	8.2	0.36
Metastatic	1	257	1.19 (0.91-1.55)	0.200	—	—
Cut-off						
≥150	3	485	1.59 (1.03-2.46)	0.036	56.2	0.102
<150	5	919	1.33 (1.14-1.56)	<0.001	0	0.505
Analysis method						
Univariate	2	430	1.16 (0.93-1.45)	0.174	0	0.778
Multivariate	6	974	1.52 (1.27-1.81)	<0.001	0	0.426

**Table 4:** Meta-analysis of the association between PLR and clinicopathological features of CCA.

Characteristics	No. of studies	No. of patients	OR (95% CI)	p	Heterogeneity	
					I^2^ (%)	Ph
Age (≥ median vs. < median)	3	669	0.82 (0.38-1.77)	0.61	70	0.03
Gender (male vs. female)	4	789	0.59 (0.44-0.80)	< 0.001	0	0.94
CA199 (>37 ng/mL vs. <37 ng/mL)	3	669	1.25 (0.92-1.70)	0.16	0	0.56
Differentiation (low vs. moderate/high)	2	442	1.05 (0.64-1.73)	0.85	0	0.90
Lymph node metastasis (pos vs. neg)	4	1194	1.16 (0.82-1.65)	0.39	0	0.63
Vascular invasion (pos vs. neg)	2	978	1.27 (0.86-1.89)	0.23	0	0.56
Postoperative complication (present vs. absent)	2	776	1.44 (0.97-2.14)	0.07	0	0.39
Postoperative mortality (present vs. absent)	2	776	1.54 (0.56-4.26)	0.41	0	0.67
Margin status (R1 vs. R0)	2	776	2.09 (1.24-3.54)	0.006	0	0.69

R0: microscopically negative resection margins; R1: microscopically positive resection margins.

## Supplementary Materials

Supplementary Materials S1: search strategies in the databases. S2: data related to this study.
