# Supplementary material for: Prognostic Significance of Platelet-to-Lymphocyte Ratio in Cholangiocarcinoma: A Meta-Analysis
Source: Biomed Res Int. 2018 Nov 14;2018:7375169. doi: 10.1155/2018/7375169 (PMC6261069; doi:10.1155/2018/7375169)
Supplement: Supplementary Materials — S1: search strategies in the databases. S2: data related to this study. [file 7375169.f1.zip › 7375169.f1/Search Strategies.doc]

1. **CENTRAL:**

#1 MeSH descriptor: [Cholangiocarcinoma] explode all trees

#2 MeSH descriptor: [Bile Duct Neoplasms] explode all trees

#3 cholangiocarcinom* or (bile duct and (cancer* or carcinom* or malignan* or neoplasm* or tumor* or tumour*))

#4 #1 or #2 or #3

#5 (PLR or platelet to lymphocyte ratio or platelet lymphocyte ratio or platelet-lymphocyte ratio).tw.

#6 #4 and #5

**2. MEDLINE:1946-**

1. exp [Cholangiocarcinoma](http://ovidsp.tx.ovid.com.nyu.pubmed007.cn:8080/sp-3.25.0a/ovidweb.cgi?&Controlled+Vocabulary=Mapping|0&Return=mapping&S=CDIHFPBAHKDDCMDJNCGKPADCLILKAA00)/

2. exp [Bile Duct Neoplasms](http://ovidsp.tx.ovid.com.nyu.pubmed007.cn:8080/sp-3.25.0a/ovidweb.cgi?&Controlled+Vocabulary=Mapping|3&Return=mapping&S=CDIHFPBAHKDDCMDJNCGKPADCLILKAA00)/

3. (cholangiocarcinom* or (bile duct and (cancer* or carcinom* or malignan* or neoplasm* or tumor* or tumour*))).mp. [mp=title, abstract, original title, name of substance word, subject heading word, keyword heading word, protocol supplementary concept word, rare disease supplementary concept word, unique identifier]

4. 1 or 2 or 3

5. (PLR or platelet to lymphocyte ratio or platelet lymphocyte ratio or platelet-lymphocyte ratio).mp.

6. 4 and 5

**3. Embase: 1974-**

1. exp [bile duct carcinoma](http://ovidsp.tx.ovid.com.nyu.pubmed007.cn:8080/sp-3.25.0a/ovidweb.cgi?&Controlled+Vocabulary=Mapping|0&Return=mapping&S=CLGOFPMAJGDDCMINNCGKCFFBFBKIAA00)/

2. exp [bile duct cancer](http://ovidsp.tx.ovid.com.nyu.pubmed007.cn:8080/sp-3.25.0a/ovidweb.cgi?&Controlled+Vocabulary=Mapping|1&Return=mapping&S=CLGOFPMAJGDDCMINNCGKCFFBFBKIAA00)/

3. exp [bile duct tumor](http://ovidsp.tx.ovid.com.nyu.pubmed007.cn:8080/sp-3.25.0a/ovidweb.cgi?&Controlled+Vocabulary=Mapping|2&Return=mapping&S=CLGOFPMAJGDDCMINNCGKCFFBFBKIAA00)/

4. (cholangiocarcinom* or (bile duct and (cancer* or carcinom* or malignan* or neoplasm* or tumor* or tumour*))).mp. [mp=title, abstract, heading word, drug trade name, original title, device manufacturer, drug manufacturer, device trade name, keyword]

5. 1 or 2 or 3 or 4

6. (PLR or platelet to lymphocyte ratio or platelet lymphocyte ratio or platelet-lymphocyte ratio).mp.

7. 5 and 6
